# Supplementary material for: MetaRibo-Seq measures translation in microbiomes
Source: Nat Commun. 2020 Jun 29;11:3268. doi: 10.1038/s41467-020-17081-z (PMC7324362; doi:10.1038/s41467-020-17081-z)
Supplement: Supplementary file 10 — Supplementary Data 7 [file 41467_2020_17081_MOESM10_ESM.zip › File2/Confidence_High_Taxonomy/222360_out.krona.html]

Javascript must be enabled to view this page.

members
magnitude
magnitudeUnassigned
count
unassigned
taxon
rank

222360\_out

22

22
superkingdom
2

22
phylum
976

22
200643
class

22
171549
order

22
family
171552

genus
838
22

22

SRS013638\_contig\_number\_1507SRS014415\_contig\_number\_1351SRS019285\_contig\_number\_64SRS043841\_contig\_number\_11077SRS044535\_contig\_number\_contig-100\_2074.98683SRS053398\_contig\_number\_10951SRS058145\_contig\_number\_2224SRS064757\_contig\_number\_502SRS065397\_contig\_number\_contig-100\_844.97276SRS077641\_contig\_number\_contig-100\_5911.114003SRS077991\_contig\_number\_5SRS097958\_contig\_number\_21398SRS103987\_contig\_number\_3294SRS103987\_contig\_number\_contig-100\_864.154548SRS104327\_contig\_number\_3171SRS143181\_contig\_number\_1647SRS144297\_contig\_number\_12681SRS144537\_contig\_number\_46582SRS144603\_contig\_number\_11765SRS144714\_contig\_number\_27487SRS146764\_contig\_number\_contig-100\_25460.79561SRS148253\_contig\_number\_12952
species
165179
